# Seismic Performance of Precast Short-Limb Shear Wall with the Bundled Connection at Different Axial Compression Ratios

**DOI:** 10.3390/ma16103870

**Published:** 2023-05-21

**Authors:** Gang Chen, Zihao Yu, Xiaohui Zhang, Hailong Yang, Qian Zhang, Jian Feng, Jianguo Cai

**Affiliations:** 1 The Third Construction Co., Ltd. of China Construction Eighth Engineering Division, Nanjing 210046, China; 2 Key Laboratory of C & PC Structures of Ministry of Education, National Prestress Engineering Research Center, Southeast University, Nanjing 211189, China

**Keywords:** precast shear wall, novel bundled connection, seismic performance, axial compressive ratio

## Abstract

In order to investigate the seismic performance of a precast shear wall with a new bundled connection under a high axial compressive ratio, three full-scale precast short-limb shear walls and one full-scale cast-in-place short-limb shear wall were manufactured and loaded under cycling loading. The results show that the precast short-limb shear wall with a new bundled connection has a similar damage mode and crack evolution to the cast-in-place shear wall. Under the same axial compression ratio, the bearing capacity, ductility coefficient, stiffness, and energy dissipation capacity of the precast short-limb shear wall were better, and its seismic performance is related to the axial compression ratio, with the increase of the axial compression ratio. The embedded bellows can limit the cracking of the wall but have little effect on the bearing capacity and stiffness degradation performance. Furthermore, the bond between the vertical steel bars extending into the preformed holes and grouting materials was demonstrated to be reliable, thus ensuring the integrity of the precast specimens.

## 1. Introduction

Concrete shear walls are commonly recognized as a key component in the resistance of lateral load in high-rise buildings, where cast-in-place concrete shear walls have been widely used thanks to their complete set of codes able to support both design and construction phases. Compared with traditional cast-in-place structures, precast shear wall structures can provide higher quality, faster construction speed, and lower labor costs, so at this stage, the construction industry in many countries promotes them [1,2]. The seismic performance of precast shear walls is largely determined by the performance of the connections between wall panels [3,4,5], although these connections are often weak parts in precast shear wall constructions [6,7]. For example, the earthquake that happened in Japan in 1995 and that happened in America in 1994 caused more damage to the connections between wall panels than other parts of the structure [8,9].

The connections of precast shear wall panels could be divided into two groups, namely the wet connection and the dry connection [10]. The wet connection can be performed by using cast-in-place concrete to connect the precast components, while the dry connection can be performed by mechanical methods. Based on the differentiation of dry and wet connections, many systems used in precast shear wall construction were investigated. Li et al. [11] tested twelve laminated concrete shear walls connected with vertical seams under low cycling loading. Results showed that the laminated concrete shear walls with well-constructed vertical seams had a similar seismic performance to the cast-in-place shear walls. Furthermore, the vertical seam could yield a larger deformation, which could improve the energy dissipation capacities of the walls. Lu et al. [12] developed a novel type of precast shear wall where a connecting beam connected the upper and lower panels into a unique integrated structure. Both the experiments and numerical simulations proved that the connecting beam could transfer the load well between panels. Meng et al. [13] proposed a method to connect the precast shear wall with a beam and tested it under numerical simulations. Results showed that the precast shear wall-beam connection had almost the same seismic performance as the cast-in-place wall-beam connection.

In order to improve the seismic performance of precast shear walls, based on the numerical simulation of concrete confined by multi-spiral hoops [14], Chen et al. proposed a new type of precast shear wall with a novel bundled connection [15]. Several specimens of the precast shear walls were designed and tested under monotonic loading while subjected to constant vertical compression. Results showed that the proposed shear wall has excellent performance, although the area at the top of the preformed holes was a weak section. Feng [16] designed and constructed several full-scale specimens, including three short-limb specimens and three specimens with a higher width-to-thickness ratio, and tested the specimens under cycling loading. Results showed that the proposed precast shear wall had good seismic performance and that the additional vertical steel bar at the preformed holes could enhance the weak sections. Liu [17] investigated the addition of a steel bar in each preformed hole, and the results showed that this measure could limit the development of cracks at the top of the preformed holes. However, the axial compression ratios of the bundled connection specimens designed in the above literature were only 0.1 and 0.2, and the seismic performance of the precast shear wall under higher axial compression ratios is not clear.

Furthermore, conventional buildings usually choose concrete shear walls with a compression ratio less than 0.2 [18,19]. The difference is that in existing high-rise buildings, the axial compression ratio of concrete shear walls is usually selected at 0.4 or higher [20]. Su and Wong [21] tested the ductility of shear wall specimens under different axial compression ratios and proved that the ductility of the shear wall is related to the axial compression ratio. Kuang and Yuen [22] used 474 sets of experimental data to investigate the influence of the axial compressive ratio on the seismic performance of concrete shear walls. The results show that the ductility of the shear wall decreases with an increase in the axial compression ratio. Hou et al. [23] tested several concrete-filled steel tube composite shear wall specimens under different axial compressive ratios. Results proved that a higher compressive ratio leads to an increase in bearing capacity and a decrease in ductility. In summary, it has been proven that the axial compressive ratio has a significant influence on the seismic performance of shear wall panels. Therefore, to clarify the seismic performance of the precast shear wall with a new type of bundled connection under a high axial compression ratio, it is necessary to further evaluate it.

In order to investigate the seismic performance of a precast shear wall with the bundled connection under a higher compressive ratio, four full-scale short-limb specimens, including three precast specimens and one cast-in-place specimen, were designed and tested under cycling loading. Where the axial compressive ratio was selected as 0.2 or 0.3. Cyclic loading tests were performed on all specimens, and the seismic performance of the specimens was evaluated from the aspects of hysteresis, skeleton, bearing capacity, stiffness, ductility, and energy dissipation. In addition, the influence of embedded and removed bellows on the seismic performance of precast shear walls was analyzed.

## 2. Materials and Methods

### 2.1. Novel Bundled Connection Concept

**Figure 1:**
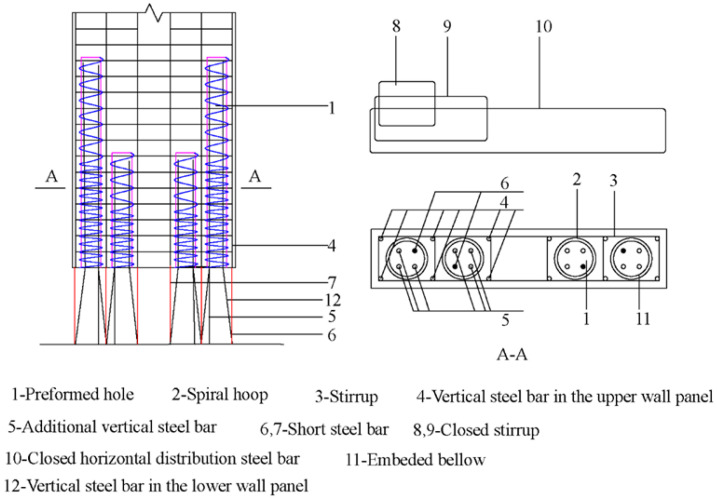
Scheme of the bundled connection method.

As shown in Figure 1, the upper and lower wall panels were connected by a connection system. Four preformed holes are present in the upper wall panel, which is covered by the embedded bellows and spiral hoops. An additional vertical steel bar is added to each preformed hole with the prerequisite of having enough anchor length. In the lower panel, three vertical rebars are divided into a group and extended into the corresponding preformed holes after two kinks. Short bars are set in a place where the vertical steel bars are kinked for the first time, thus replacing the kinked vertical bars in supporting the closed stirrups. Then, the preformed holes are filled with grouting materials. After the curing of the grouting materials, the upper and lower wall panels are assembled into one integrated structure.

This construction method avoids the construction accuracy problem of inserting each steel bar into its corresponding preformed hole in the existing vertical reinforcement connection technology. The preformed hole is large enough to facilitate the lap of vertical steel bars, solving the problem of precise positioning required in the existing vertical reinforcement connection technology and making the on-site construction convenient and efficient. In addition, the pre-buried connection bars at the top of the lower wall panel, together with the vertical steel bars, are bundled and extended into a preformed hole set at the bottom of the upper wall, effectively reducing the crack width. The pre-buried spiral hoops in the hole can effectively restrain the concrete in the compression zone, improving its strength and the grip force of the lapped vertical steel bars. Combined with the stress gradient characteristics of the vertical steel bars in the inner and outer preformed holes, the structural details of the precast shear wall with the different heights are proposed to reduce the crack width at the end of the preformed holes. Structural measures such as short and additional vertical steel bars are added to further ensure the reliability of the vertical reinforcement lap connection and overall structural performance.

### 2.2. Specimens Design

To comprehensively understand the seismic performance of a precast shear wall with a bundled connection under a high axial compressive ratio, experiments were carried out on four full-scale specimens subjected to cycling loading. When the specimen was designed based on the commonly used secondary seismic rating, 0.3 is the upper limit of the axial compression ratio with only the structural edge members at the bottom wall limb section [24]. If the axial compression ratio continues to increase, it is necessary to design the restrained edge members, and additional requirements exist for longitudinal vertical steel and hoop bars. Furthermore, 0.2 is the commonly used axial compression ratio parameter in engineering and research [21,25,26,27].

Specimen XJA was a cast-in-place specimen, and specimen YZA-1 was the corresponding prefabricated specimen with the same reinforcement, which was mainly used to compare the effects of the connection method on the seismic performance of the structure. Specimen YZA-2 was used to investigate the effect of the bellows on the seismic performance by only screwing out the bellows after the concrete was poured as compared to YZA-1. Specimens YZA-1 and YZA-3 differed only in axial compression ratio, and the structural measures were identical, in order to analyze their seismic performance under the action of different axial compression ratios. The detailed information on the specimens is shown in Table 1.

**Figure 2:**
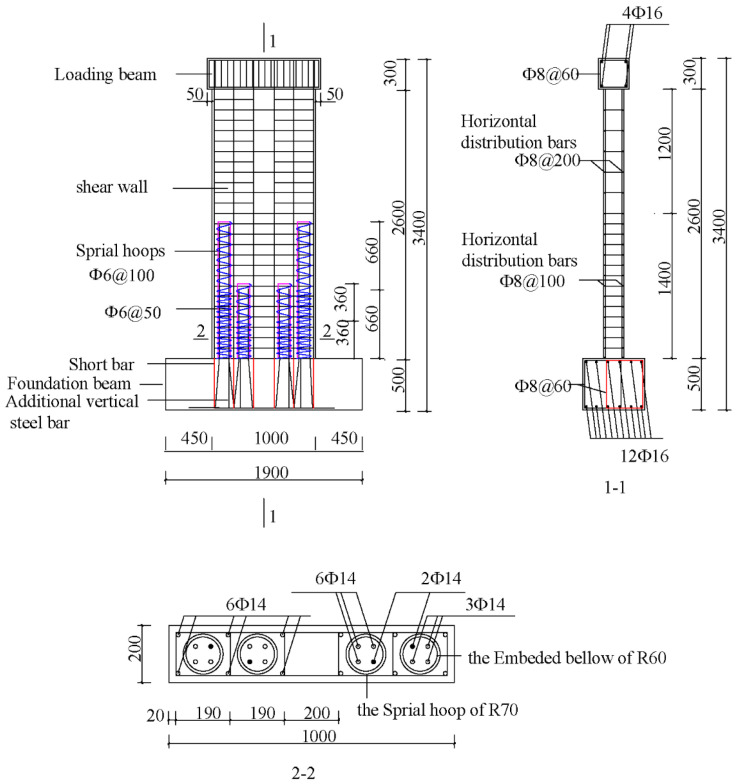
Configuration of YZA-1, YZA-2, and YZA-3.

**Figure 3:**
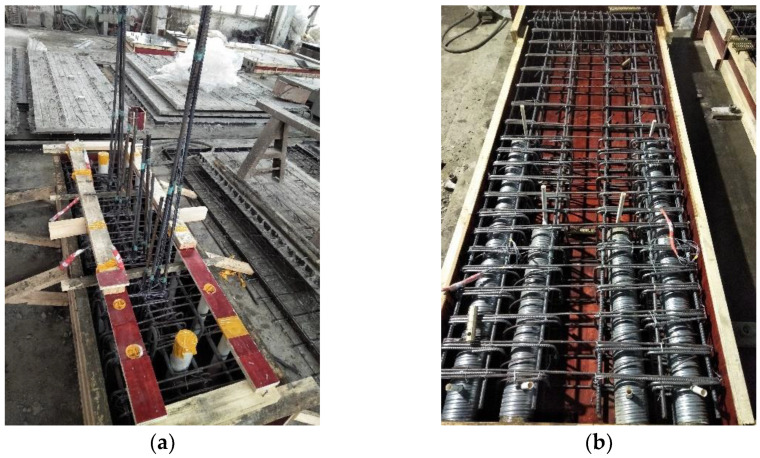
Illustration of the bundled connection details. (**a**) The steel bars in the lower wall. (**b**) The upper wall.

**Figure 4:**
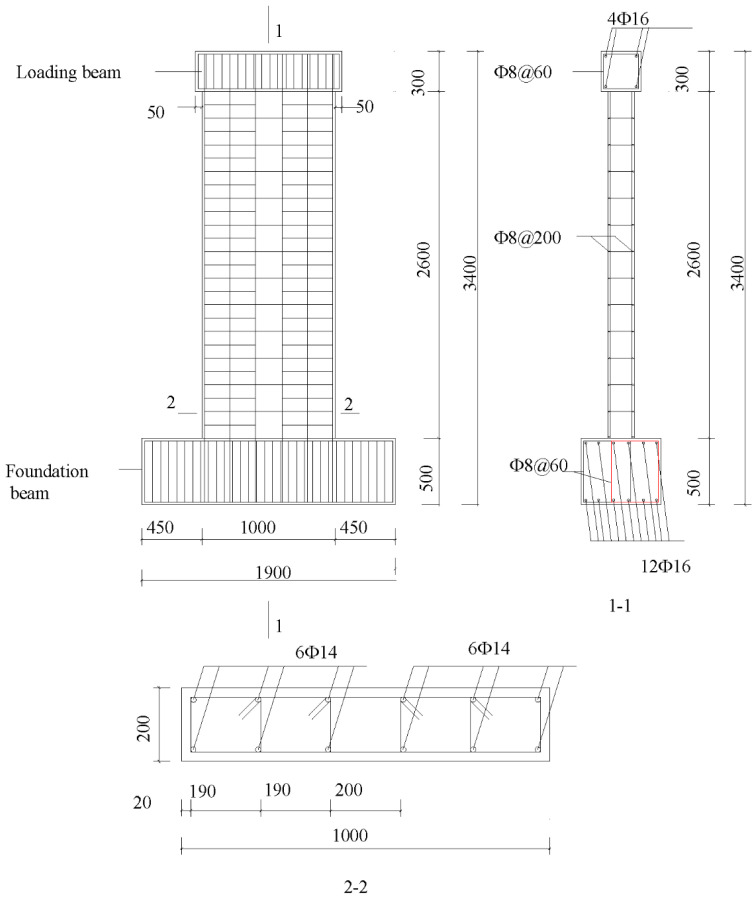
Configuration of XJA.

The size of each precast shear wall specimen was 1000 mm × 2600 mm × 200 mm, as shown in Figure 2. The ratios of width-to-thickness of all specimens were 5, i.e., short-limb shear walls. The loading beam was fixed on top of the shear wall to provide horizontal and vertical loads. The foundation beam had a length, width, and height of 450 mm, 1900 mm, and 450 mm, respectively. The height of the outer preformed holes was 1320 mm, while one of the inner preformed holes was 720 mm. The length of the vertical steel bars extending into the preformed holes was 50 mm shorter than the height of the corresponding preformed holes. The anchor length of the additional steel bars in the preformed holes was 480 mm. The bundled connection details are shown in Figure 3. The cast-in-place specimen XJA had the same dimensions as the precast shear wall specimens. The detailed layouts of the steel bars of XJA are shown in Figure 4. Furthermore, the precast wall, the loading beam, and the foundation beam are made of C40-grade concrete, with a compression strength equal to 40 MPa according to the Chinese Code for Concrete Structures [28]. The steel bars with diameters of 8 mm, 12 mm, and 14 mm used in the specimen were HRB400, and the steel bar with a diameter of 6 mm was HPB300.

### 2.3. Material Properties

All materials were tested, including reinforcement bars, concrete, and grouting materials, at the Nanjing Dadi Laboratory. When manufacturing each precast shear wall specimen, three 150 mm × 150 mm ×150 mm cube concrete samples were manufactured and cured under the same boundary conditions as the shear wall specimen. When filling the preformed holes in each shear wall specimen with grouting material, three 40 mm × 40 mm × 160 mm cylinder samples of grouting materials were manufactured and cured under the same boundary conditions as the shear wall specimen. The concrete and grouting material are both provided by Nanjing Xipu Cement Engineering Group Co., Ltd. (Nanjing, China), and the grouting material was required to use non-shrinkage cement-based grouting material. These cubes and cylinders were tested during the experimental period to evaluate the compression strength of the concrete, and the corresponding elastic moduli were obtained by the empirical formula based on the compression strength [29]. Furthermore, three samples of steel bars were tested under tensile loading to obtain the yield and ultimate strengths [30]. The corresponding properties of concrete and grouting material are listed in Table 2, and Table 3 gives the measured yield and ultimate strength of the steel bar.

### 2.4. Experimental Test Setup and Loading System

**Figure 5:**
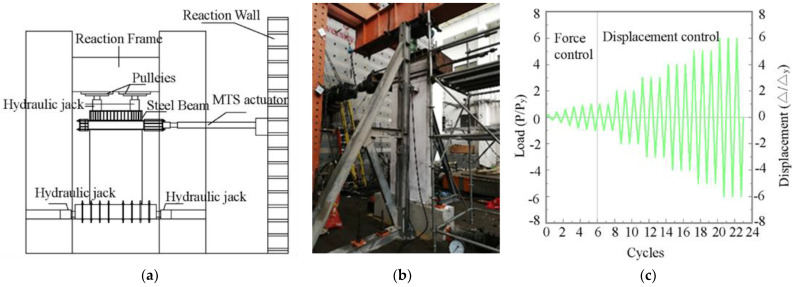
Test up and loading system. (**a**) Schematic of the test setup. (**b**) Test-up in the lab. (**c**) Loading system.

The schematic diagram of the related loading equipment is shown in Figure 5a. The loading setup consists of the reaction frame, the vertical loading device, the horizontal loading device, the bottom fixing device, and the lateral supporting device, as shown in Figure 5b. The axial force was realized by the hydraulic jack and the pulleys between the reaction frame, with the hydraulic jack ensuring an axial vertical force. The horizontal force was realized by the MTS actuator, which was connected to the reaction beam and the reaction wall. The foundation beam was fixed to the wall.

The mixed load of force and displacement was used in the test, and the loading process is shown in Figure 5c. It should be noted that *P*, *P*_y_, and Δ_y_ stand for the horizontal force, the yield horizontal force, the displacement, and the yield displacement, respectively. Before yielding, the load process was controlled by a force that increased by 40 kN for each cycle. After the yielding of the specimen, the load process changed to be controlled by displacement. Each amplitude increased by Δ_y_ and had three cycles. The tests were stopped when the bearing capacities of the specimens dropped to 85% of the peak load or when the specimens could no longer bear the axial load. 

## 3. Results

### 3.1. The Evolution of Cracks

**Figure 6:**
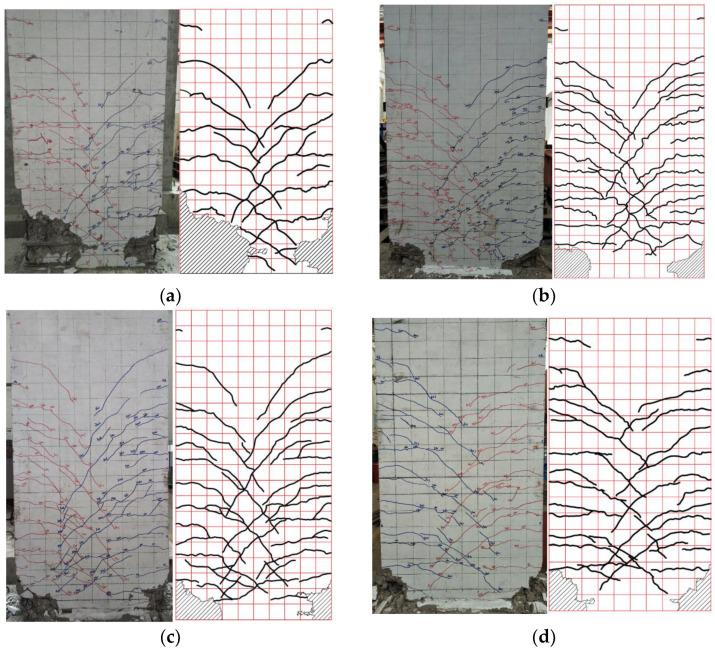
Crack distribution and damage modes of specimens. (**a**) XJA. (**b**) YZA-1. (**c**) YZA-2. (**d**) YZA-3.

In order to study the cracking behavior of shear walls, the failure modes of the specimen were analyzed. The final damage and crack distribution of all specimens are shown in Figure 6. It can be seen that the precast and cast-in-place specimens have similar failure modes and crack evolution.

At the initial stage of loading, the specimen was in an elastic state, and no cracks were present. With continued loading, multiple horizontally distributed cracks appeared in the lower part of the wall. As displacement increases, the crack tip continues to expand forward, and some horizontal cracks begin to transform into oblique cracks. At this time, the length extension of horizontal cracks is roughly in the range of 180–400 mm. Finally, the height of the horizontal crack gradually expands upward and is roughly stable below half the height of the wall.

After entering the yield state, it is mainly the evolution of oblique cracks. Full development of oblique cracks occurs during 2–3 times yield displacement the range of oblique crack extension length is roughly 140–800 mm. Finally, the cracks on both sides intersect in the middle of the wall. At this stage, the number of cracks in the wall no longer increases, and there is no significant change in the shape and length of the cracks.

At the late stage of loading, the specimen was finally destroyed, and the concrete at the bottom of the compressed zone was severely crushed and spalled. All the prefabricated specimens had buckling and fractures in the reinforcement in the outer pre-drilled holes. For XJA, the crack width at the bottom was expanded to 3 mm when the concrete in the compressed zone started to spill. Compared with the precast specimen, specimen XJA showed a larger area of concrete spalling on the left side of the bottom of the compression zone, with a spalling height of 600 mm, and the steel bar on the right side also has obvious buckling deformation.

In order to clarify the effect of the variation in axial compression ratio and the condition of the bellows on the wall cracking, all specimens were compared and analyzed. From the final damage mode of the specimens, it can be seen that different numbers of cracks were produced on both sides of the wall. However, the YZA series has more cracks, smaller crack widths, and better integrity than the XJA. This indicates that the bundled, connected precast shear walls have better seismic capacity.

The crack expansions of specimens YZA-1 and YZA-2 were similar, which indicated that removing the bellows had little effect on the crack expansion of the precast shear walls. Compared with YZA-1, the cracks in YZA-3 were more fully extended, and the number of cracks was lower, which indicates that the higher axial compression ratio can better limit crack propagation. In addition, during the crack extension, the initial fracture cracks of XJA appeared roughly at the wall heights of 170 mm and 380 mm in the tensile zone. While the initial fracture cracks of all prefabricated specimens appeared near the top of the reserved holes and eventually evolved into the main cracks.

### 3.2. Hysteresis Loops and Envelope Curves

**Figure 7:**
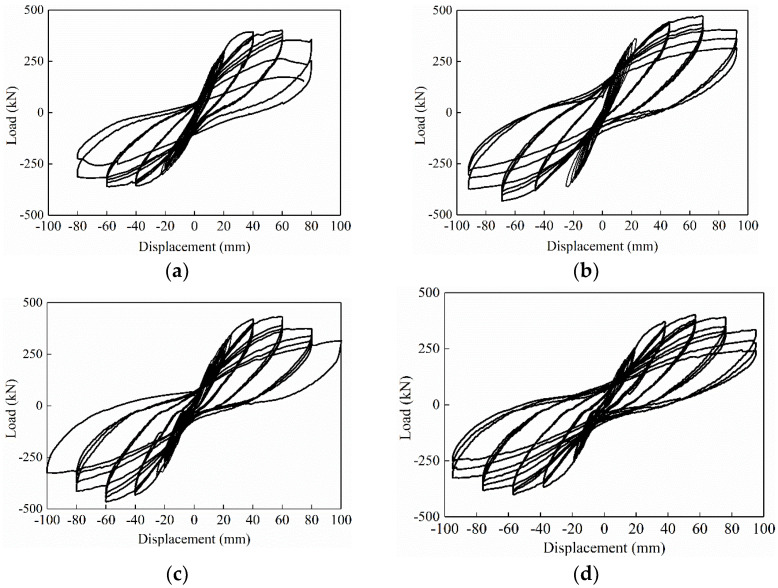
Hysteresis curves of specimens. (**a**) XJA. (**b**) YZA-1. (**c**) YZA-2. (**d**) YZA-3.

The load-displacement hysteresis curves of the specimens are shown in Figure 7. It can be seen that the hysteresis curves of all the specimens are relatively full, and there is no obvious pinch phenomenon. Before the specimen cracks, the load-displacement curve varies approximately linearly, and there is basically no residual deformation after unloading. With displacement loading, the specimen cracked, the area of the hysteresis loop gradually increased, and residual deformation occurred after unloading. Entering the yield state, the hysteresis loop area continues to increase, the slope of the hysteresis curves gradually decreases, and the residual deformation increases after unloading. When the displacement was loaded to the last control stage, the bearing capacity of specimen XJA decreased rapidly, while the YZA series decreased more slowly, which may be due to the more reinforcement in the lap zone of the prefabricated specimen. 

Compared with XJA, the hysteresis loop area of all YZA was significantly increased, and the ability to resist deformation was also significantly improved, which indicates that the proposed bundled connection precast shear wall has good energy dissipation capacity. Furthermore, although the ultimate displacement of specimen YZA-2 is slightly higher than that of YZA-1, the fullness of the hysteresis curve is smaller than that of YZA-1, indicating that the removal of the bellows has little effect on the seismic performance of the specimen. Compared with YZA-3, the peak load of YZA-1 is higher than YZA-3 and has a higher hysteresis curve fullness, which indicates that the proposed bundled connection precast shear wall has better seismic performance under a higher axial compression ratio.

**Figure 8:**
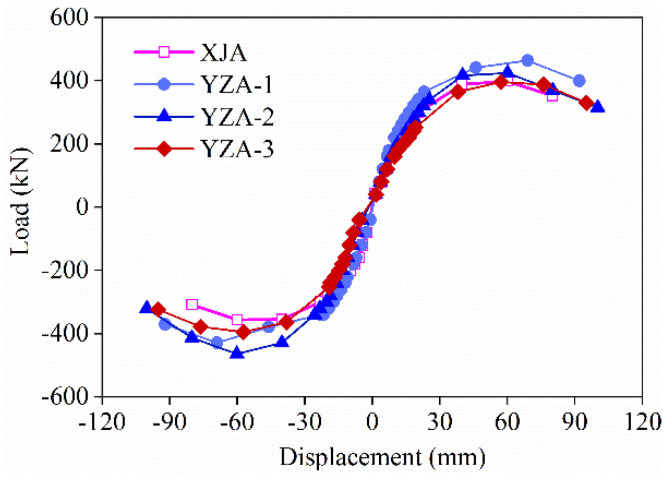
Skeleton curves of specimens.

Figure 8 shows the skeleton curves of all specimens under cyclic loading. It can be seen that all the skeleton curves have the same trend; especially before specimen cracking, the curves were consistent, indicating that the stiffness difference between the specimens at this stage was small. With displacement loading, the growth rate of the bearing capacity of the specimen gradually increases, and the wall begins to crack, which is due to the fact that cracks need to accumulate a certain amount of energy to be generated. When the displacement exceeds 30 mm, the bearing capacity growth rate gradually decreases, which is due to the multiple cracks generated in the wall at this stage and the accumulated energy dissipated. At the later stage of loading, the crushing of concrete and the fracture of steel bars lead to the reduction of the ultimate bearing capacity to below 85% of the peak load.

It can be seen from Figure 8 that the specimen still maintains a high bearing capacity from the yield stage to the ultimate stage. Therefore, all specimens show good ductility, especially the precast series specimens. The load-displacement skeleton curves of all precast specimens are higher than those of cast-in-place specimens XJA, indicating that the proposed bundle connection precast short-limb shear walls have better deformation capacity. And it can significantly increase the load capacity and ultimate displacement of the specimen.

When the positive displacement of the skeleton curves is greater than 60 mm, the bearing capacity of YZA-1 is higher than that of YZA-2, indicating that the presence of bellows can improve the bearing capacity of the precast short-limb shear wall. Compared with YZA-3, the positive load carrying capacity of YZA-1 increases by more than 20% compared to YZA-3 when the displacement exceeds 19 mm, and the reverse bearing capacity improves less, which indicates that increasing the axial compression ratio could also improve the bearing capacity of precast short-limb shear walls.

### 3.3. Carrying Capacity

Table 4 lists the cracking load *F*_cr_, yield load *F*_y_, and peak load *F*_p_ of all specimens. The cracking loads and yield loads correspond to the loads when the first crack occurs and the vertical steel bar in the tensile zone yields, respectively [31]. Table 4 shows that the *F*_y_ and *F*_p_ of all precast specimens are higher than those of cast-in-place specimens XJA. The *F*_cr_ values of YZA-2 and YZA-3 were the same as those of the precast specimen XJA, while the average *F*_cr_ of YZA-1 was about 42% improved compared with that of XJA. The results show that when the axial compression ratio is increased, initial precompression stress is generated in the concrete, which can retard the appearance of initial cracks. Similarly, the presence of corrugated tubes makes the concrete within the height of the preformed hole more integral, which also retards the initial cracks. The difference between the *F*_y_ and *F*_p_ values of specimens YZA-2 and YZA-1 is small, which indicates that although the bellows tube added to YZA-1 can limit the cracking of the wall cracks, it has little effect on the bearing capacity. 

Compared with YZA-3, the *F*_cr_, *F*_y_, and *F*_p_ of YZA-1 were higher than those of YZA-3, and the average values of *F*_y_ and *F*_p_ of YZA-1 were 4.3% and 12.9% higher than those of YZA-3, respectively. It shows that the values of *F*_cr_, *F*_y_, and *F*_p_ increase with the increase of the axial compression ratio, which also means that the cracking of the wall can be limited and the load-bearing capacity of the precast short-limb shear wall can be improved by increasing the axial compression ratio. In addition, the ratio of tensile strength to yield strength for all specimens was in the range of 1.13–1.25, indicating that the specimens had sufficient safety reserves.

### 3.4. Ductility

Displacement angle and ductility coefficient are important indicators reflecting the seismic performance of the structure, which are calculated using Equation (1) and Equation (2), respectively [31]. Table 5 lists the yield displacement Δ_y_, yield displacement angle *θ*_y_, ultimate displacement Δ_u_, ultimate displacement angle *θ*_u_, and ductility coefficient *μ* of all specimens.

(1) θ=Δ/H

(2) $$\mu =\Delta_{u}/\Delta_{y}$$

where Δ_y_ and Δ_u_ are the horizontal displacements of the loading point at the yield and failure of the specimen, respectively. H is the height from the loading point to the bottom of the shear wall, which is 2750 mm.

It can be seen from Table 5 that, compared with the cast-in-place specimen XJA, the ultimate displacement Δ_u_ of all precast short-limb shear walls YZA is higher than that of XJA and at least increases by about 15%. In addition, the average value of the ductility coefficient *μ* of the prefabricated specimen YZA-1 was slightly larger than that of XJA, while the average values of μ of YZA-2 and YZA-3 were smaller than those of YZA-1 and XJA, and the μ value of YZA-3 decreased more obviously. It shows that the *μ* value is mainly related to the axial compression ratio of the sample and increases with the increase of the axial compression ratio. 

In summary, although the proposed new bundle connection can better improve the deformation capacity of precast short-limb shear walls, the variation of the ductility coefficient *μ* value is unstable. Therefore, it is recommended to set bellows or increase the axial compression ratio to improve the ductility and deformation capacity of the specimens for precast short-limb shear walls with bundled connections. It is worth noting that the ultimate displacement angle *θ*_u_ of all prefabricated specimens at failure meets the minimum limit requirement of 1/120 [24].

### 3.5. Stiffness Degradation

**Figure 9:**
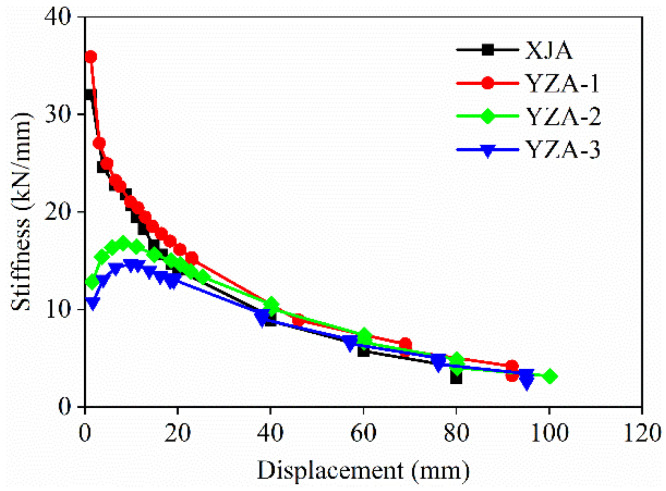
Stiffness degradation of specimens.

The stiffness degradation curves of all specimens are shown in Figure 9. It can be seen that the stiffness of samples XJA and YZA-1 shows a downward trend in the early stage of loading, while YZA-2 and YZA-3 show an upward trend. This may be due to the sliding of specimens YZA-2 and YZA-3 during the initial loading process, resulting in a smaller initial stiffness. When the displacement developed to about 20 mm, the stiffness degradation curves of all specimens began to slowly decrease, and the trend was almost the same.

The results of specimens XJA and YZA-1 were discussed as an example. Before the specimen yields, the stiffness drops sharply, mainly due to the damage caused to the specimen by the development of micro cracks within the specimen. After the specimen yields, the main crack gradually forms, and the development of some small cracks tends to be stable. The stiffness degradation rate slows down at this stage.

In addition, the initial stiffness of YZA-1 was slightly larger than that of XJA, indicating that the proposed prefabricated shear wall with bundled connection has excellent stiffness degradation performance. The descending section of YZA-2 is almost the same as that of YZA-1, while the stiffness of YZA-3 is lower than that of YZA-1 during the whole loading process, which indicates that increasing the axial compression ratio can improve the stiffness of the precast shear wall, but the setting of bellows in the wall has little effect on the stiffness degradation performance. Table 6 lists the cracking stiffness *K*_cr_, yield stiffness *K*_y_, peak stiffness *K*_p_, and ultimate stiffness *K*_u_ for all specimens.

### 3.6. Energy Dissipation Capacity

**Figure 10:**
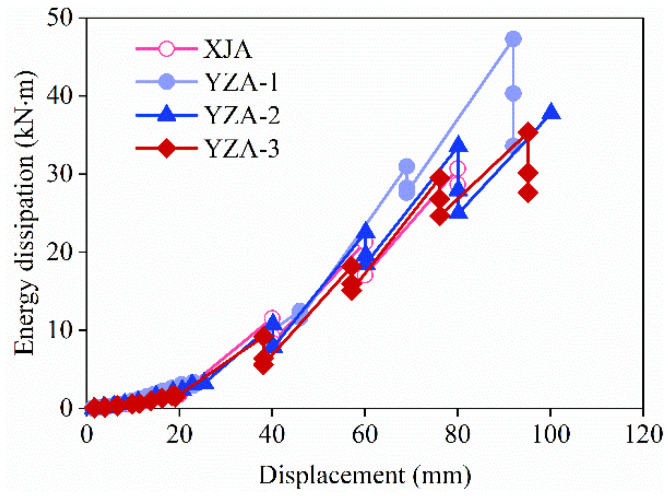
Cumulative energy dissipation of specimens.

The energy dissipation capacity is also an important parameter to evaluate the seismic performance of the structure, and the stronger the energy dissipation capacity, the better the seismic performance. The energy dissipated by the specimens was determined by the area enclosed by the load-displacement hysteresis curves obtained under cyclic loading. Figure 10 shows the energy dissipation-displacement curves for all specimens.

It can be seen that during the force loading phase, the specimen displacements were small, the energy dissipation curves of all specimens almost overlapped, and the dissipated energy was small. As the loading continues, the specimen begins to crack and further evolve, and the energy dissipation gradually increases. Entering the displacement cyclic loading phase, the energy dissipation capacity of each level increases significantly with the increase in displacement. However, under the same horizontal displacement loading, due to the decrease in stiffness of the specimens, the ability of energy dissipation was weakened in each cycle, and the degree of weakening gradually increased with the increase in displacement.

In addition, the energy dissipation of YZA-1 was significantly greater than the other three specimens, which indicates that the energy dissipation capacity can be improved by setting corrugated tubes in the wall and increasing the axial compression ratio.

### 3.7. Strains in Steel Bars

**Figure 11:**
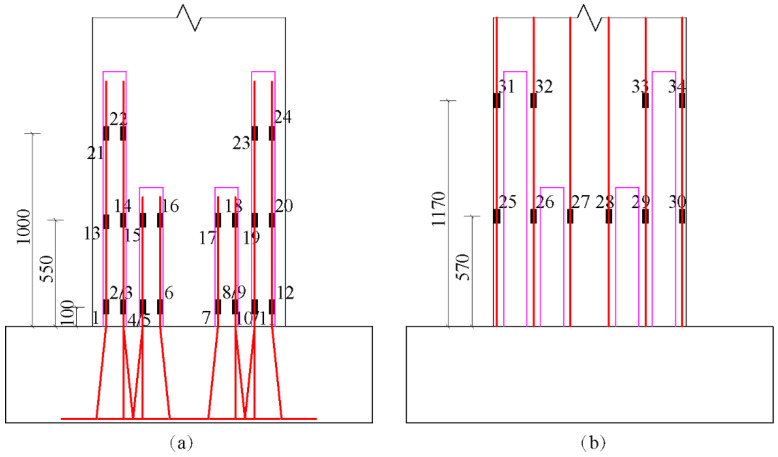
The layouts of strain gauges: (**a**) on the vertical steel bars extending into the preformed holes; (**b**) on the vertical steel bars in the wall.

The transfer performance of the vertical reinforcement in the precast shear wall preformed hole is very important for the connection of the upper and lower wall panels. Therefore, in order to study the reliability of the bundled connections under a high axial compression ratio, the strain data of the vertical reinforcement and the wall under cyclic loading were analyzed. The results of specimen YZA-1 were discussed as an example. The layout of strain gauges is illustrated in Figure 11.

**Figure 12:**
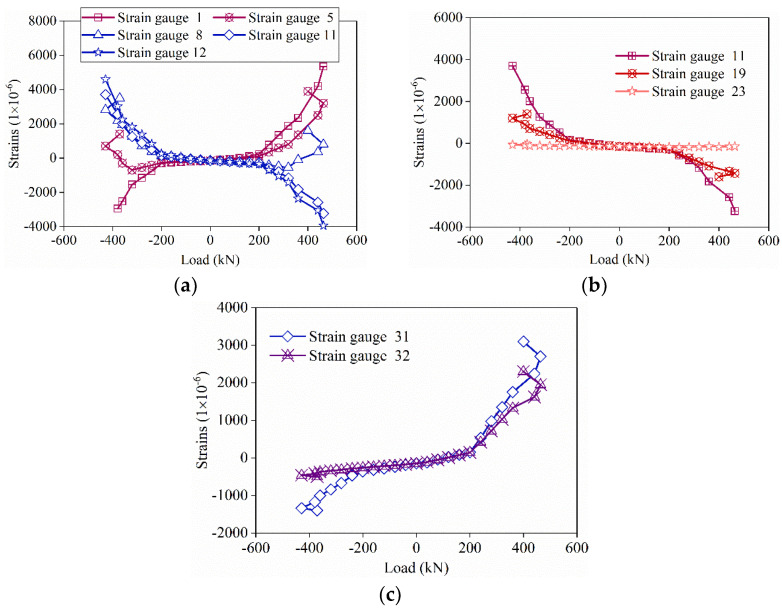
Load-strain curves for vertical reinforcement of specimen YZA-1. (**a**) Different vertical reinforcement at the same height. (**b**) Same vertical reinforcement at different heights. (**c**) On the vertical steel bars in the wall at the same height.

Figure 12 shows the relationship between the horizontal force and the strains of the vertical steel bars in the specimens. Before the horizontal load was applied, the strains of all gauges were negative since the specimen was only in axial compression. Gauges 1 and 5 were installed on the same height of vertical steel bars extending into different preformed holes. When the load was between −200 kN and 200 kN, the strains of gauges 1 and 5 were approximately equal, and the values were small. When the load exceeded 200 kN, the strains of gauges 1 and 5 rose rapidly. The reason may be that the specimens started to crack when the load exceeded 200 kN, and the deformation of the specimen increased rapidly. With the increase in load, the strain of gauge 1 was always higher than that of gauge 5. The maximum strain of gauge 1 was 5500 × 10^−6^ in positive load and −3000 × 10^−6^ in negative load, which was higher than the yield strain of the HRB steel bar with a diameter of 14 mm, 2250 × 10^−6^. This phenomenon means that gauge 1 yielded both positive and negative loads. The maximum of gauge 5 was 4000 × 10^−6^ in positive load, meaning that gauge 5 was yielded in positive load. The same phenomenon could be observed in the strains of gauges 8 and 12. The strain value of gauge 11 was between that of gauge 8 and that of gauge 12. 

As shown in Figure 12b, gauges 11, 19, and 23 were installed at different heights on a vertical steel bar in a preformed hole. When the load was less than 200 kN, the strains of gauges 11, 19, and 23 were close to each other. When the load exceeded 200 kN, the strains in gauges 11 and 19 rose, with gauge 11 rising more rapidly. The gauges 11 and 19 were yielded in the end while the strain of gauge 23 still kept a negative value during the test. This phenomenon showed that the strains on vertical steel bars at higher positions were higher. The reason may be that a distance was needed to transfer the force between the steel bars and the grouting material. This aspect indicated that the vertical steel bars extending into the preformed holes connected well with the grouting material.

As shown in Figure 12c, when the load was less than 200 kN, the strains of gauges 31 and 32 were almost equal. When the load exceeded 200 kN, the strains of gauges 31 and 32 rose rapidly, and the strain of gauge 31 was higher than that of gauge 32, which may be due to the cracking of the specimen. Under the peak load, gauges 31 and 32 yielded in positive load but not in negative load. These phenomena show that the bundled connection can better transfer the stress of the steel bar, thus ensuring the continuity of the force transmission of the prefabricated shear wall.

## 4. Conclusions

The seismic performance of the new bundled connection precast shear walls under a high axial pressure ratio was investigated by cyclic load tests, and some conclusions were obtained. The precast and cast-in-place specimens have similar damage modes and crack evolutions. Before the precast specimen yields, the crack mainly develops in the horizontal direction. After yielding, it is mainly the evolution of oblique cracks. During the loading period of 2–3 times the yield displacement, the oblique crack was fully developed, and the extension length range was about 140 mm to 800 mm. In addition, increasing the axial compression ratio can limit the crack propagation of precast shear walls. 

Compared with the cast-in-place specimen, the hysteresis loop area and deformation resistance of the precast shear wall with bundled connection were significantly increased, and the bearing capacity of the precast shear wall can be significantly improved by increasing the axial compression ratio. The embedded bellows can limit the cracking of the wall but have little effect on the bearing capacity. The ductility coefficient *μ* is related to the axial compression ratio of the specimen and increases with the increase in the axial compression ratio. The stiffness and energy dissipation capacity of the precast shear wall increase with the increase of the axial compression ratio, and the embedded bellows have little effect on the stiffness degradation performance. The bundled connection can better transfer the stress of the steel bar, thus ensuring the continuity of the force transmission of the prefabricated shear wall.

## Figures and Tables

**Table 1:** Specimen variations.

Specimen	Type	Removal of Corrugated Tubes	Axial Compressive Ratio
XJA	Cast-in-place		0.3
YZA-1	Precast	No	0.3
YZA-2	Precast	Yes	0.3
YZA-3	Precast	No	0.2

**Table 2:** Properties of concrete.

Property	Concrete	Grouting Material
Average compression strength (MPa)	42.7	66.8
Elastic modulus (GPa)	33.3	31.5

**Table 3:** Mechanical properties of reinforcement bars.

Steel Grade	Diameter (mm)	Yield Strength *f*_y_ (MPa)	Ultimate Strength *f*_u_ (MPa)
HPB300	6	380	615
HRB400	8	430	645
HRB400	10	445	660
HRB400	14	450	640
HRB400	16	435	640

**Table 4:** Load capacities of the specimens at the feature points.

Type	Loading Direction	*F*_cr_/kN	*F*_y_/kN	*F*_p_/kN
XJA	+	120	330	398
	−	120	302	356
	Average	120	316	377
YZA-1	+	180	378	464
	−	160	345	429
	Average	170	362	446
YZA-2	+	120	374	425
	−	120	389	464
	Average	120	381	444
YZA-3	+	120	366	396
	−	120	328	395
	Average	120	347	395

**Table 5:** Displacement and ductility values of specimens.

Type	Loading Direction	Δ_y_/mm	*θ*_y_	Δ_u_/mm	*θ*_u_	*μ*
XJA	+	27.20	1/101	80	1/34	2.94
	−	23.50	1/117	80	1/34	3.40
	Average	25.35	1/108	80	1/34	3.17
YZA-1	+	28.37	1/97	92	1/30	3.24
	−	25.98	1/106	92	1/30	3.54
	Average	27.18	1/101	92	1/30	3.39
YZA-2	+	31.99	1/86	100	1/28	3.13
	−	33.53	1/82	100	1/28	2.98
	Average	32.76	1/84	100	1/28	3.05
YZA-3	+	38.63	1/71	95	1/29	2.46
	−	32.05	1/86	95	1/29	2.96
	Average	35.34	1/79	95	1/29	2.71

**Table 6:** Equivalent stiffness of specimens at different stages (unit: kN/mm).

Type	Loading Direction	*K*_cr_	*K*_y_	*K*_p_	*K*_u_
XJA	+	18.84	12.15	6.62	3.08
	−	28.64	12.83	5.92	2.75
	Average	23.74	12.49	6.27	2.92
YZA-1	+	23.90	13.33	6.71	3.40
	−	22.36	13.27	6.20	3.06
	Average	23.13	13.23	6.45	3.23
YZA-2	+	20.38	11.69	7.06	3.15
	−	13.62	11.59	7.71	3.20
	Average	17.00	11.64	7.39	3.17
YZA-3	+	18.10	9.47	6.93	6.91
	−	11.83	10.25	3.47	3.40
	Average	14.96	9.86	5.20	5.16

## Data Availability

Data sharing is not applicable, all data obtained from this study are already given in the article.
